# Driving Research and Advocacy for Healthy Infant and Toddler Diets: The Infant and Toddler Foods Research Alliance

**DOI:** 10.1111/mcn.70013

**Published:** 2025-03-13

**Authors:** Alexandra Chung, Jennifer McCann, Emma Esdaile, Naomi Hull, Andrea Schmidtke, Sally MacKay, Penelope Love, Rachel Laws, Catharine A. K. Fleming

**Affiliations:** ^1^ Department of Nutrition, Dietetics and Food Monash University Melbourne Australia; ^2^ Institute for Physical Activity and Nutrition, School of Exercise and Nutrition Sciences Deakin University Geelong Australia; ^3^ Centre for Childhood Nutrition Research Queensland University of Technology Brisbane Australia; ^4^ Sydney School of Public Health The University of Sydney Sydney Australia; ^5^ Food for Health Alliance Melbourne Australia; ^6^ Department of Epidemiology and Biostatistics School of Population Health University of Auckland Auckland New Zealand; ^7^ School of Health Sciences Western Sydney University Sydney Australia

**Keywords:** early childhood, first‐foods, food, infant, nutrition, toddler

## Abstract

Early childhood (0–36 months) is a critical time for the development of healthy dietary behaviours. This paper describes the establishment of the Infant and Toddler Foods Research Alliance in Australia and New Zealand, along with the development of the Alliance's priorities to guide research and advocacy activities for improved nutrition, health and well‐being outcomes in early childhood. The multi‐disciplinary Alliance includes a membership of academics, practitioners and advocates working in the fields of infant and toddler food and nutrition across Australia and New Zealand. The Alliance undertook a priority setting process across a series of member meetings with identified priorities subsequently refined by a core membership working group. Three priority themes, along with three cross‐cutting impact areas were identified. The priority themes include commercial foods and milks for infants and toddlers; health and care settings and systems; and support for parents and carers. The cross‐cutting impact areas include building evidence, translating evidence, and advocacy. This provides a framework to guide research, practice and advocacy, identify research gaps, and advance action to improve nutrition, health and well‐being outcomes for infants and toddlers.

## Summary

- The Infant and Toddler Foods Research Alliance was formed in Australia and New Zealand in 2022 to drive research and action to improve nutrition, health, and well‐being outcomes in early childhood.
- The Alliance membership includes academics, practitioners, and advocates working together to build and translate evidence and lead advocacy for improved infant and toddler nutrition.
- The Alliance has prioritised three themes: commercial foods and milks for infants and toddlers, health and care settings and systems, and support for parents and carers.

## Graphical abstract

The Infant and Toddler Foods Research Alliance brings together researchers and health professionals across Australia and New Zealand with an interest in infant and toddler food regulation, child development, and interventions to better support optimal dietary and feeding outcomes for infants and toddlers.

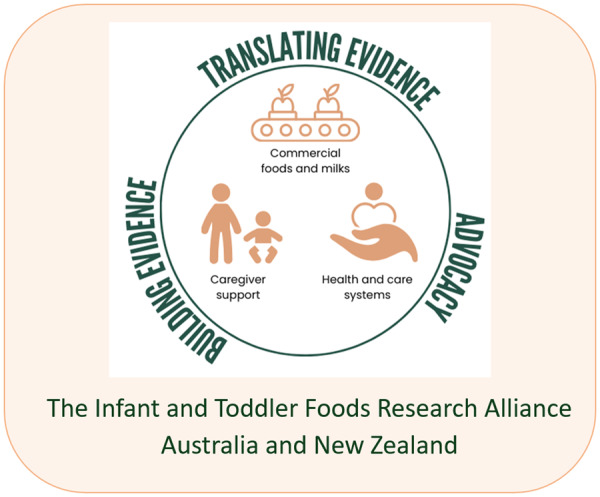

## 1 Introduction

Early childhood (0–36 months) is a critical time for growth and development. This important developmental period is a window of opportunity for establishing healthy dietary behaviours that can set the foundations for lifelong health and well‐being and reduce the risk of chronic disease outcomes later in life (Ogata and Hayes 2014). Dietary behaviours in early life have implications for growth and health, with the quality of a child's diet more important at this time of their life than at any other time (United Nations Children's Fund 2020). Poor nutrition in the early years of life impacts survival into adulthood, increases the risk of non‐communicable diseases and influences educational outcomes and opportunities to earn an income (United Nations Children's Fund 2020). Yet, the United Nations child malnutrition estimates indicate increasing levels of all forms of malnutrition, including undernutrition (wasting, stunting and underweight) and overweight (World Health Organization 2020). The Food and Agriculture Organization (FAO) reported that globally in 2022, an estimated 148.1 million children under 5 years of age (22.3%) experienced stunting, 45 million (6.8%) experienced wasting, 37 million (5.6%) experienced overweight and fewer than 50% of babies met the 6‐month recommendation for exclusive breastfeeding. This data suggests that globally we are not on track to meet the 2030 nutrition targets outlined in the Sustainable Development Goals (FAO, IFAD, UNICEF, WFP, and WHO 2023). While wasting is not an issue in Australia and New Zealand (Global Nutrition Report 2024), overweight and obesity in children has remained steady at 25% in Australia (ages 2–17 years) since 2007, and around 32% in New Zealand since 2011/12 (ages 2–14 years) (Australian Institute of Health and Welfare 2020; Ministry of Health 2024).

The health and economic costs of childhood malnutrition cannot be underestimated (Black et al. 2017; Skouteris et al. 2021; World Health Organization 2018). Obesity is a crude measure of one form of malnutrition, however its global impacts are extensive with an estimated 254 million children under 5 years of age predicted to be living with obesity by 2020 (Ling et al. 2023). The individual and societal costs of such childhood obesity are consequently rising, costing approximately $45 billion USD per year. The World Health Organization (WHO) Commission on Ending Childhood Obesity recommends actions that support increased duration of breastfeeding and healthy diets in early childhood (World Health Organization 2016), with improved diet quality in early life identified as one of the most promising interventions for obesity prevention in the first 2 years of life (Baur and Garnett 2018).

Nutrition for healthy growth and development is not merely about the absence of disease. Nutrition in early life is a prime opportunity to promote the development of healthy dietary behaviours that support lifelong health and well‐being. Dietary intake and food preferences of young children are influenced by parental and family factors, social and early childhood education and care settings, as well as the broader food environment shaped by food availability, accessibility and marketing (Ravikumar et al. 2022; Scaglioni et al. 2018). Families worldwide face economic, political, market, social and cultural barriers to providing nutritious diets to infants (0–12 months) and toddlers (12–36 months) (Fenta et al. 2023; Turner et al. 2020). Many of these barriers are not experienced equally across society, exacerbating dietary and health inequities. Infants and toddlers, mothers and other family members are frequently exposed to foods of low nutritive value, including commercial complementary foods and processed foods containing added and free sugars (e.g., from fruit‐based commercial baby/toddler foods), salt and saturated fats (United Nations Children's Fund 2019), shaping dietary behaviours. Once formed, these dietary patterns and preferences for commercial and processed foods tend to continue from early childhood into the preschool (3–5 years) and later childhood years (Lioret et al. 2015; Luque et al. 2018). In general, infants and young children do not need specific commercial foods, as they can meet their needs through the consumption of healthy family foods.

Food and nutrition literacy starts in early childhood, with the experiences of what (diet quality) and how young children are fed. Responsive feeding is the seventh recommendation of the WHO Guideline for Complementary Feeding, defined as ‘feeding practices that encourage the child to eat autonomously and in response to physiological and developmental needs, which may encourage self‐regulation in eating and support cognitive, emotional and social development’ (World Health Organization 2023a). Evidence based, responsive feeding recommendations to support families with nurturing feeding practices and promote autonomous, self‐regulated eating within children should underpin the current research translation agenda to ensure optimal infant feeding development occurs for infants and toddlers (Pérez‐Escamilla et al. 2021). Promoting healthy diets in early childhood also presents an important opportunity to achieve health equity (Skouteris et al. 2021). Young children's dietary behaviours are socioeconomically patterned such that children experiencing socioeconomic disadvantage are more likely to consume unhealthy food and drinks compared to children with higher socioeconomic position (Chung et al. 2018). These inequities in young children's diets have implications for the development of obesity and socioeconomic inequities in obesity (Chung et al. 2018), reinforcing the importance of healthy diets in early childhood.

## 2 Why Does Australia and New Zealand Need a Research Alliance Focused on Infants and Toddlers?

The Infant and Toddler Food Research Alliance (The Alliance) was founded by a group of Australian researchers in early 2022 in response to identified evidence gaps around early childhood feeding and long‐term feeding trajectories and an increase in availability of commercial foods for infants and toddlers. To reflect that Australia and New Zealand have a bi‐national food regulatory system, Alliance membership was recently extended to New Zealand. To our knowledge, there is no other Australia/New Zealand‐wide research collaboration with the same focus. Initial invitation for membership was through personal networks and known researchers in the field. The Alliance includes a multidisciplinary membership of researchers, practitioners, clinicians and implementers, situated in academic, nongovernment and health promotion organisations. It remains a goal of The Alliance executive committee to ensure cultural diversity amongst Alliance members by inviting Aboriginal and/or Torres Strait Islander, Māori and culturally diverse people working in this field to join the Alliance. To uphold the integrity of the Alliance and be completely impartial to commercial influence in all policy, advocacy and research conducted under the Alliance name we have a Conflict of Interest policy that requires potential members to declare any potential conflicts, consistent with the WHO's Declaration of Interests for WHO Experts. Membership is underpinned by shared values to protect and improve early childhood nutrition and a desire to overcome barriers created by the siloed nature of research in this field. The aim of The Alliance is to increase collaboration within and between institutions and disciplines by building a coalition that will grow and generate momentum for policy and practice that promotes optimal infant and toddler nutrition, health and well‐being. We are encouraged by the notion that when academics, clinicians and civil society work together as a coalition to reframe issues and mobilise resources, political will can be generated (Baker et al. 2018).

There is limited published research from Australia and New Zealand containing comprehensive data on energy, protein and micronutrient intakes in early childhood—this gap warrants further research. Instead, the literature tends to focus on dietary intake in comparison to dietary recommendations. In Australia, the diets of infants (0–12 months) and toddlers (12–36 months) fall well short of recommended guidelines. Australia's National Infant Feeding Survey reported breastfeeding initiation rates of 96%, declining to just 15% of infants exclusively breastfed at 5 months, with 60% of infants receiving a mix of breastmilk and commercial milk formula (CMF) at 5 months of age (Australian Institute of Health and Welfare 2011). Statewide data from Victoria showed similar rates of breastfeeding initiation (95.6%) but also indicated that 30% of term, breastfed babies were given infant formula in hospital (Yuen et al. 2022). National data indicates that only 20% of Australian children aged 2–3 years consume the recommended servings of vegetables each day (Australian Bureau of Statistics 2023). At the same time, discretionary food contributes 30% of total daily energy intake for children 2–3 years, and 52% of 2‐ to 3‐year‐olds exceed the recommended intake of free sugars (Australian Institute of Health and Welfare 2018). Longitudinal data from the Melbourne Infant Feeding Activity and Nutrition Trial Programme found a sharp decline in dietary quality from infancy to toddlerhood, reporting that only 3% of 18‐month‐olds consumed the recommended amount of vegetables compared to 96% of 9 month olds (Spence et al. 2018).

Growing Up in New Zealand is a large contemporary birth cohort comprising babies born 2009/2010 that has provided the first nationally generalizable information on indicators of early life nutrition (Morton et al. 2015). Among this cohort, breastfeeding initiation rates were 97%, and 18% of infants were exclusively breastfed to around 6 months (Castro et al. 2017). Around 40% of the cohort were introduced to complementary foods too early (≤ 4 months) (Ferreira et al. 2023). At 9 months, respectively for boys and girls, only 38% and 36% were having fruit daily and only 31% and 34% were having vegetables daily (Castro et al. 2022). At 2 years and 4.5 years respectively, 39% and 55% of the cohort were consuming the number of servings of vegetables and fruit recommended by the national food and nutrition guidelines (Gontijo de Castro et al. 2022). The assessment of whole diet quality at 9 and 24 months using dietary indexes measuring the cohort's adherence to the national guidelines indicated on average a poor adherence to the guidelines. The study has also reported the consistent identification at 9, 24, and 54 months of a dietary pattern with high consumption of processed foods and foods with high content of sodium, sugar, and unhealthy fats (Gontijo de Castro et al. 2023).

Whilst our focus is on infant and toddler diets, we recognise the significance of the first 2000 days, the period from conception to age 5, for optimal child health and development. In Australia the importance of early life has been recognised with the first 2000 days prioritised in the Government's Early Years Strategy 2024–2034 (Commonwealth of Australia 2024). In New Zealand, the NZ Health Strategy 2023 emphasises that ‘support during the early years sets us up for the best possible health and well‐being outcomes later in life’. The Child and Youth Strategy 2024–27 prioritises supporting children and their families and whanau in the first 2000 years (Te Kawanatanga o Aotearoa New Zealand Government 2024). Despite this, there are gaps in evidence and policy. A recent rapid review using the Innocenti Framework (UNICEF 2018) found significant evidence gaps for the first 2000 days (Laws et al. 2022) and early childhood is not widely accounted for across the spectrum of public health (Esdaile et al. 2019; Esdaile et al. 2022; Esdaile et al. 2022; Love et al. 2022). As a result, current environments do not adequately support optimal infant and toddler feeding, and there is a need for coordinated action across a range of settings and sectors internationally, nationally, and locally (Laws et al. 2022).

We also acknowledge the significance of early childhood education and care (ECEC) settings and their important role in supporting nutrition throughout early childhood. In Australia, the National Nutrition Network (NNN)—Early Childhood Education and Care exists with a mission to promote healthy, sustainable food environments within Early Childhood Education and Care (ECEC) settings. There is strong membership crossover between the NNN and The Alliance, and thus to avoid duplication this Alliance has not included ECEC settings as a priority.

## 3 Equity and a Child Rights Approach to Early Childhood Nutrition

The Alliance's work in early childhood nutrition is underpinned by principles of equity, social justice and child rights. The UN Convention on the Rights of the Child (UNCRC) applies to all children whatever their background (religion, race, family of origin) (Article 2) and states that they have a right to live a full life and that governments have the responsibility to ensure they develop healthily (Article 6). Children also have a right to good quality health care, nutritious food and an environment that enables them to stay healthy (Article 24). Ratification of the UNCRC means that Australia and New Zealand have a duty to ensure that all children in Australia and New Zealand enjoy the rights set out in the UNCRC. This includes protecting children from unhealthy food environments which undermine their basic human right to adequate nutrition and healthy food (United Nations Children's Fund 2019). This requires implementation of a suite of policies and programmes by governments to create and maintain accessible, affordable, sustainable, healthy food systems and address the underlying social, economic and political factors that influence health and health equity.

## 4 The Priorities of the Infant and Toddler Food Research Alliance

**Figure 1:**
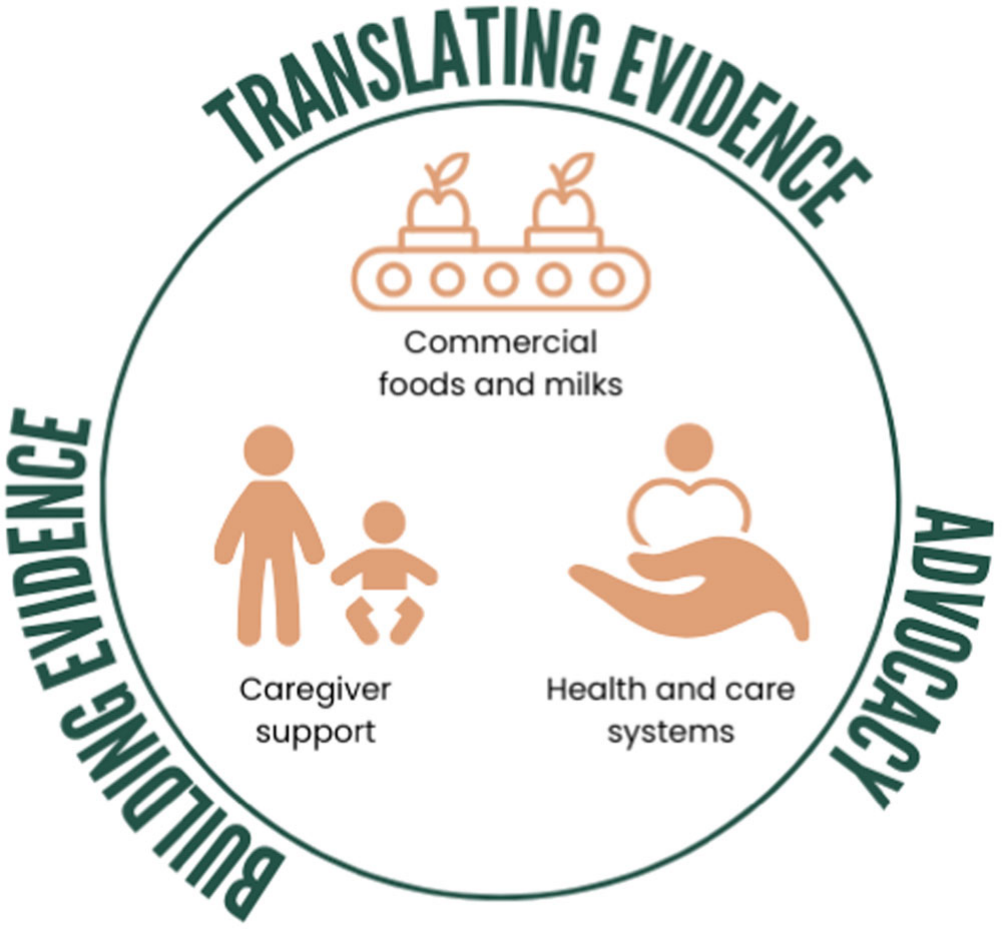
Visual representation of the priority themes and cross‐cutting impact areas of the Infant and Toddler Foods Research Alliance.

The ultimate goal of The Alliance is for young children to meet their dietary needs through healthy family foods within health, care, and food systems that support them to do so. To achieve this goal, members of The Alliance initiated a priority setting process in 2023 to identify priority areas to guide collaborative work across The Alliance. First a meeting was held among The Alliance Executive Committee members where it was agreed to identify and communicate our shared priorities. This idea was put forward at a member meeting where an invitation to join a working group was extended to all members. A working group was formed and participants identified topic areas of importance. Through a series of working group meetings, these were synthesised into three priority themes and three cross‐cutting impact areas. Finally, the priority themes and cross‐cutting impact areas were put to the entire membership for discussion. With support received from the broader membership, these now form a framework to guide our collective work, building and translating evidence and advocating for improved nutrition for infants and toddlers. The priority themes and cross‐cutting impact areas are articulated below and visualised in Figure 1.

**Priority themes**

A. Commercial foods and milks for infants and toddlers
B. Health and care settings and systems
C. Support for parents and carers

**Cross‐cutting impact areas**

1. **Building evidence**. Build a strong independent evidence basis surrounding infant and toddler foods and feeding. Strengthen evidence through targeted research and collaboration across disciplines and sectors.
2. **Translating evidence**. Facilitate evidence translation into practice and the public domain, tailored according to different contexts. This includes the work of academic institutions to translate evidence and best practice guidelines for application in real world settings as well as the role of governments to facilitate best practice through policies and initiatives.
3. **Advocacy**. Lead and support advocacy for change that is based on research and stimulates a renewed focus/prioritisation on healthy and sustainable nutrition for infant and toddlers.

## 5 Priority A: Commercial Food and Milks for Infants and Toddlers

Breastmilk substitutes (BMS) including CMFs (infant formula, follow‐on formula and toddler milks) and commercially produced foods for infants and toddlers, including readymade meals, snacks and purees are part of a billion‐dollar global market (GlobeNewswire 2022). There is an extensive range of CMFs available on Australian supermarket shelves (McCann et al. 2021). Over the past few decades there has been a rise in use of CMFs despite World Health Organization guidance recommending young children not to consume them (WHO Comp Feeding). CMFs are heavily promoted by the baby food industry through sophisticated marketing and political practices that impede actions to protect and promote breastfeeding (Baker et al. 2021; Baker et al. 2023). This transition within the ‘first‐foods system’ is due to urbanisation, devaluing of the care economy and changing roles of women, medicalisation of birth and ineffective regulation of marketing of CMFs and associated products (Baker 2020; Gribble et al. 2023). While the Alliance is not able to counteract urbanisation and devaluing the care economy, it can work to advocate for the protection of breastfeeding through full implementation of the WHO Code of Marketing of Breastmilk Substitutes (including breastmilk substitutes, toddler milks as well as foods marketed for infants and toddlers) and the subsequent World Health Assembly resolutions (The Code) in Australia and New Zealand. In Australia throughout 2023, The Alliance participated in consultations and made submissions to the Australian Competition and Consumer Commission (ACCC) regarding the Infant Nutrition Council's application for reauthorisation of the existing voluntary agreement that has been shown to be ineffective (Allen and Clarke Consulting 2023). The decision is pending at time of writing.

Like CMFs, commercial foods for infants and toddlers are heavily marketed to parents and caregivers (Chung et al. 2023; McCann et al. 2021). The number of foods for infants and toddlers available on the Australian market doubled between 2013 and 2018 (McCann et al. 2021). Commercial practices drive the availability, accessibility and desirability of infant and toddler foods, undermining breastfeeding and optimal feeding practices for infants and toddlers (Chung et al. 2022). These products are promoted to parents and carers as nutritious, safe and convenient (Brunacci et al. 2023; Chung et al. 2024; McCann et al. 2021; McCann et al. 2021; Simmonds et al. 2021). However, parents' perceptions of these foods and their expectations of strict government regulation do not align with reality (The Royal Children's Hospital Melbourne 2022).

Most commercial infant and toddler foods in Australia do not align with National Dietary Guidelines or WHO recommendations for composition and promotion of foods for infants and young children (Dunford et al. 2024; Scully et al. 2024). These products frequently contain high levels of added and free sugars, offer minimal nutritional value and are often highly processed with a sweet taste profile. The World Health Organization recommends that infants and young children do not consume foods and beverages high in sugar, salt and trans fats (World Health Organization 2023a) however these foods are readily available and promoted to parents and caregivers. For example, in Australian supermarkets 80% of first foods for infants are sweet, fruit‐based purees, highly processed extruded puffs are commonly promoted as snack foods for toddlers and 40% of all products contain free sugars (Moumin et al. 2020). Products packaged in squeeze pouches are common and most are sweet and uniformly pureed, limiting children's exposure to the diversity of flavours and textures needed to support oral‐motor development and the transition to family foods (Brunacci et al. 2023). Australian infants and toddlers frequently consume these ready‐made foods (Brunacci et al. 2023; Gascoyne et al. 2021). As part of ongoing advocacy for healthy diets for infants and young children, the Alliance will advocate that future reviews of national early childhood feeding and nutrition guidelines align with current evidence and international best practice, including the WHO Guidelines for Complementary Feeding of Infants and Young Children (World Health Organization 2023a) and WHO recommendations for composition and promotion of food products for infants and young children (World Health Organization Regional Office for Europe 2022).

In New Zealand, more than two‐thirds (69%) of commercial infant and toddler foods have sweet flavours, and sweet vegetables are more common than bitter ones. Most products are smooth or pureed (62%) and while textural complexity increases along the recommended age gradient for savoury meals, this is less common in fruit‐based meals and breakfast foods which are more likely to be smooth purees (Padarath et al. 2020). Over half of the commercially available snack foods for infants and toddlers in New Zealand contain added sugars, and iron content is generally low except in fortified cereals (Katiforis et al. 2021).

Frequent exposure to added and/or free sugars in the first years of life can reinforce the innate preference that infants have for sweet foods (Fidler Mis et al. 2017) with taste preferences persisting throughout life and influencing dietary patterns in adulthood (Birch et al. 2007). Given the high amount of added and free sugars in many commercial infant and toddler food products (Brunacci et al. 2023; McCann et al. 2021; Scully et al. 2023), this remains a key area of concern for early childhood feeding.

Globally, awareness and discussion regarding the impact on long term health of ultra‐processed foods is increasing, as there is mounting evidence on the negative health implications of diets high in UP foods and drinks in early childhood (Childs and Sibson 2023; Elizabeth et al. 1955; Machado et al. 2022; Oliveira et al. 2022). This is important in the context of well‐established evidence that dietary intakes and preferences formed in early childhood track into later childhood and even adulthood (Lioret et al. 2013; Spence et al. 2018). It is important to understand the role and impact of ultra‐processed foods in early childhood feeding, and The Alliance will work to contribute to this evidence base and to support best practice policies that protect and promote child health.

The WHO has urged governments to reduce the exposure and impact of unhealthy food marketing (World Health Organization 2010; World Health Organization 2023c). Specific guidance in the Nutrient and Promotion Profile Model: supporting appropriate promotion of food products for infants and young children 6–36 months in the WHO European Region, provides detailed recommendations on the nutritional composition, labelling and promotion of foods for infants and toddlers (WHO Regional Office for Europe 2022).

Commercial foods for infants and young children should support health, growth and development and should not undermine breastfeeding or optimal infant and young child feeding practices. The Alliance will continue to advocate for comprehensive government‐led regulation to ensure CMFs and commercial foods for infants and toddlers meet international best practice and national dietary guidelines. This includes adequate nutritional composition and appropriate textures of foods for infants and toddlers, accurate labelling and restrictions on marketing and promotion that mislead or overstate the health, nutrition and developmental benefits of products or their ingredients. To ensure compliance, regulations must include provisions for monitoring and enforcement (Bero et al. 2019).

## 6 Priority B: Health and Care Settings and Systems

In 2019, Australian Health Ministers agreed to a goal of 50% of infants exclusively breastfed to 6 months of age by 2025 (COAG Health Council 2019). However, policies that appropriately protect and support breastfeeding have not been prioritised by governments in Australia and New Zealand (Hull et al. 2018; Hull et al. 2017; Smith 2007). A significant barrier to achieving this goal is practices within health and hospital settings. One Victorian study found that approximately 30% of healthy term infants have received infant formula before they leave hospital (Yuen et al. 2022). High rates of introduction to commercial milk formulas may be due to the low uptake of UNICEF's Baby Friendly Health Initiative (BFHI) with only approximately 30% of maternity services accredited in Australia (World Breastfeeding Trends Initiative Australia 2023). BFHI has been shown to positively impact breastfeeding rates in Australia (Esbati et al. 2020; Smith et al. 2018) through the mechanisms of education and training for hospital staff and increased awareness of supportive practices, such as keeping mothers and babies together, adherence to the WHO Code, staff education and professional development on infant feeding. Globally, the BFHI has been found to have a positive impact on breastfeeding outcomes in the short, medium and long term, with evidence of a dose–response relationship such that exposure to more of the ten BFHI steps is associated with improved breastfeeding outcomes (Pérez‐Escamilla et al. 2016). If mothers and babies go home already utilising infant formula with a lack of appropriate support, establishing and maintaining breastfeeding can be challenging (Pérez‐Escamilla et al. 2016; Smith et al. 2018). More localised research is needed to establish an effective business case to ensure the political will and appropriate level of funding so that BFHI accreditation can be effectively implemented nationwide.

The National Breastfeeding Strategy for Aotearoa aims to increase the exclusivity and duration of breastfeeding in Aotearoa. In 2023, 16 of 19 Health New Zealand (Te Whatu Ora) locations met the Baby Friendly Hospital Initiative of at least 75% of infants receiving only breastmilk throughout their stay in the maternity service. The New Zealand Breastfeeding Authority reports national infant feeding data at discharge from the maternity service. In 2023, 77.06% of infants were exclusively breastfed, 2.27% fully breastfed, 16.39% partially breastfed and 3.98% artificially fed (Baby Friendly Aotearoa New Zealand 2023).

In high‐income countries, mothers and their infants utilise primary healthcare (PHC) services more frequently during the early years of life than at any other point in childhood, with many visits being unrelated to illness (Hayes et al. 2019; Ou et al. 2017). Consequently, PHC providers including general practitioners and child and family health nurses are well‐positioned to monitor growth and development and offer parental support regarding child feeding. However, evidence suggests that PHC providers need additional assistance in effectively monitoring growth trajectories and communicating with parents around growth and fostering healthy eating habits (Rossiter et al. 2023). Studies involving child and family health nurses in Australia have found that while most routinely measure height and weight, promote breastfeeding and recommend water as the main drink (for those > 12 months old), while limiting sugar‐sweetened beverages, less time is spent discussing best practices for formula feeding, increasing fruit and vegetable intake and limiting discretionary foods (Cheng et al. 2020; Laws et al. 2015). There is no evidence on how, or whether, child and family health nurses and other practitioners are communicating with caregivers about how to navigate commercial foods for infants and toddlers. Key barriers identified by PHC providers include concerns about parental receptiveness to healthy lifestyle advice and parental motivation to enact change. Ensuring PHC practitioners are kept up to date with evidence on commercial foods for babies and toddlers is likely important to enable conversations about child feeding and growth that reflect the current food environment. Further research is needed to identify appropriate strategies and interventions to achieve this goal. The Alliance is prioritising research cohesion and collaboration in this area to promote practice‐ready and evidence‐based support for the health and care settings and systems.

## 7 Priority C: Enabling Parents and Carers

The Alliance's final priority is to enable parents and carers, and the work of our members in this priority has two streams. The first is to engage in research to identify the needs of parents and carers and evaluate policies, programmes and services offered to parents and carers. The second is to advocate to governments to implement policies, programmes and services that meet these needs. Parents and caregivers have a primary role in providing adequate nutrition in early childhood and the greatest impact on a child's eating behaviours during these early years (Laws et al. 2022). Yet the needs of parents and caregivers are often overlooked within early years policies, despite parent and caregiver health and well‐being being vital to their ability to care for their children (Kitson et al. 2022). Maternal nutrition in particular is crucial due to its effects on the child's development in utero and the mother's own health and well‐being, which in turn influences her capacity to breastfeed and provide adequate care (World Health Organization 2023b). The paternal influence on dietary behaviours and child health throughout early life is also an area of growing interest (Kuswara et al. 2025). The mental health of parents and caregivers should be first and foremost a priority, yet for some that do access these types of services, they may feel a sense of failure for asking for this type of advice (De Sousa Machado et al. 2020). However, there is a need to reduce the stigma around accessing this type of support. High‐quality evidence to support the integration of multi‐component breastfeeding education and counselling into existing service‐delivery systems with a focus on continuity of support across antenatal and postnatal services is urgently required. At present there is a gap in current understanding of what breastfeeding support is available and accessed by families (Laws et al. 2022).

An approach that starts from the ground up is required to empower parents and caregivers to navigate feeding their infants and toddlers during this vulnerable stage of life. In Australia, the efficacious INFANT programme aims to support parents with healthy eating and active play from the start of life through group peer support and a mobile phone app (Campbell et al. 2016). Clear, independent, conflict of interest free, evidence‐based resources with advice on family foods, responsive feeding and transitioning to the complementary feeding period, including how to navigate commercial foods for infants and toddlers, are essential to ensure healthy food provision in early childhood (McCann et al. 2024). Lack of regulation and policy in Australia is enabling an imbalance of information, with misinformation and targeted marketing towards parents pervasive (Jones et al. 2023). Increasing the rates of breastfeeding and dissemination of conflict free, evidence‐based resources on young child feeding is needed (Errico et al. 2024). In addition, infant and toddler food packaging is heavily branded with on‐pack marketing aimed at both children and caregivers. To mitigate the impact on young children's diets from the negative impact of food marketing, strong government regulation is necessary (Chung et al. 2023).

In New Zealand, Healthy Babies Healthy Futures is a collaboration between Te Whatu Ora—Health New Zealand, NGOs and academics (Healthy Babies Healthy Futures 2024). The collaboration supports the health and well‐being of pregnant mothers and parents of children under 4 years of age in Auckland by promoting breastfeeding, healthy eating and keeping active. The programme offers a text support service, lifestyle coaches, nutrition and well‐being courses. Evaluation shows that almost all participants made at least two positive changes to eating behaviours and many (79%) felt more confident about cooking healthy meals (Healthier Lives Implementation Network 2024).

Governments can prioritize support for parents and caregivers by integrating parent and caregiver support into early years policies and expanding access to successful, evidence‐based early childhood nutrition resources and programmes. Child health and development screening checks relating to diet intake and health behaviours should be universally available and standardised in Australia and New Zealand to ensure all parents and caregivers are being provided with up‐to‐date, personalised information when feeding children in the first few years of life. Underscoring these approaches is the need for policies that ensure all families have access to healthy, affordable, safe and culturally appropriate food for their young children.

## 8 Conclusion

The Alliance comprises a multidisciplinary membership committed to building and translating evidence and leading advocacy efforts to promote and protect healthy dietary behaviours for all infants and toddlers throughout Australia and New Zealand. Early childhood represents a critical period for developing healthy diets and building the foundations of healthy growth and development. Priority areas for infant and toddler nutrition include supporting parents and carers, creating health‐promoting environments across health and care settings, and ensuring the products and practices of commercial infant and toddler food and milk industries do not undermine infant and toddler health. Government‐led initiatives that align with international guidance and best practice will be crucial to advancing action across each of these priority areas to ensure lifelong health and well‐being.

## Author Contributions

C.F. and J.M. initiated the Infant and Toddler Foods Research Alliance. AC proposed the idea for this article. A.C. and E.E. prepared the original draft. A.C. and J.M. coordinated the critical review and revision of subsequent drafts. All authors contributed to the writing and revising of the article. All authors have read and approved the final manuscript.

## Conflicts of Interest

E.E. is employed at the CCHR, funded by the Queensland Children's Hospital Foundation philanthropic grant from Woolworths staff and customer donations. The funders played no role in the design, implementation or reporting of the research undertaken at the CCHR.
